# Bodycomposition, bone mineral density and serum adipokines in juvenile idiopathic arthritis with previous glucocorticoid therapy

**DOI:** 10.1186/1546-0096-12-S1-P142

**Published:** 2014-09-17

**Authors:** Kati P Markula-Patjas, Helena Valta, Sture Andersson, Heli Viljakainen, Outi Mäkitie

**Affiliations:** 1 Children's Hospital, Helsinki University Central Hospital and University of Helsinki, Helsinki, Finland; 2 Paediatric Research Centre, University of Tampere and Tampere University Hospital, Tampere, Finland; 3 Folkhälsan Research Center, Helsinki, Finland

## Introduction

Patients with juvenile idiopathic arthritis (JIA) are prone to alterations in bone health, body composition and adipokines.

## Objectives

To evaluate areal bone mineral density (BMD), body composition and serum leptin and adiponectin levels in patients with JIA and healthy controls.

## Methods

Study cohort comprised 50 patients with JIA (34 girls, median age 12.7 and range 7.4-17.4 years, median disease duration 6.3 and range 2.0-15.1 years) previously exposed to systemic glucocorticoids for a minimum of three months (range 0.2-12.5 years). Study protocol included clinical examination, bone age determination, BMD and body composition measurements with dual X-ray absorptiometry (DXA), and assessment of fasting serum leptin and adiponectin. Comparison with age- and gender-matched healthy controls (n=88) was performed.

## Results

Height, weight, BMI, pubertal stage, fat mass and lean mass were similar. Patients had lower Z-scores for whole body and bone-age-corrected lumbar spine BMD (p<0.001 and 0.009). Their whole body BMC to lean mass ratio was lower. Serum leptin and adiponectin were similar between groups even when adjusted for age and fat mass, and no correlations with disease activity occurred. Patients (n=16) with low LS BMD (≤-1.0 SD) were shorter, lighter and had lower BMI than those with normal BMD. Their lean mass for height and bone area for height were lower. In the low BMD group BMC to lean mass ratio was also decreased when adjusted for gender and height (p=0.002).

## Conclusion

Patients with JIA exposed to systemic GCs had normal stature, body composition and serum leptin and adiponectin levels, but reduced whole body and lumbar spine BMD. Among the patients those with lowest BMD had delayed growth, but also evidence of sarcopenia and osteopenia.

## Disclosure of interest

None declared.

